# Body Composition as a Predictor of Toxicity and Prognosis in Patients with Diffuse Large B-Cell Lymphoma Receiving R-CHOP Immunochemotherapy

**DOI:** 10.3390/curroncol28020126

**Published:** 2021-03-23

**Authors:** Jiaxun Guo, Panpan Cai, Pengfei Li, Cong Cao, Jing Zhou, Lina Dong, Yan Yang, Qijia Xuan, Jingxuan Wang, Qingyuan Zhang

**Affiliations:** 1 Department of Medical Oncology, Harbin Medical University Cancer Hospital, Harbin 150040, China; gjxsmile0920@163.com (J.G.); Ctwopan@163.com (P.C.); godlovecc1220@126.com (C.C.); zhoujing71189@163.com (J.Z.); a17853293086@163.com (L.D.); yangjy0717@126.com (Y.Y.); xuanqijia@126.com (Q.X.); 2 Radiology Department, Harbin Medical University Cancer Hospital, Harbin 150040, China; lipengfei197942@hotmail.com

**Keywords:** diffuse large B-cell lymphoma, immunochemotherapy, survival, sarcopenia, toxicity

## Abstract

Background: Our study measured the body composition of Diffuse large B-cell lymphoma (DLBCL) patients receiving rituximab with cyclophosphamide, doxorubicin, vincristine and prednisone (R-CHOP) regimen by computed tomographic (CT) and assessed their correlation with treatment-related toxicity and other adverse outcomes. Methods: We retrospectively analyzed 201 DLBCL patients who underwent pre-treatment abdominal CT examination. CT images were used to assess body composition metrics at the third lumbar vertebrae including fat tissues and muscle. Based on the skeletal muscle area (SMA) and density (SMD), skeletal muscle index (SMI), skeletal muscle gauge (SMG = SMI × SMD) and lean body mass (LBM) were calculated. Also analyzed were the toxicity, adverse events and survival. Results: We found that SMG, SMD, SMI and LBM were correlated with any grade 3–4 toxicity, dose reduction, hospitalization or termination of the treatment due to immunochemotherapy and worse survival. However, multivariate analysis demonstrated SMG [progression-free survival (PFS): hazard ratio (HR), 2.889; 95% CI, 1.401–5.959; *p* = 0.004; overall survival (OS): HR, 2.655; 95% CI, 1.218–5.787; *p* = 0.014] was the best predictor of poor prognosis. Conclusions: SMG, SMD, SMI and LBM were identified as predictors of adverse reactions and poor survival. SMG was an innovative and valuable indicator of immunochemotherapy toxicity and other adverse outcomes. Additionally, it can be used to individualize antineoplastic drug dosing.

## 1. Introduction

In adults, the most common subtype of non-Hodgkin’s lymphoma is Diffuse large B-cell lymphoma (DLBCL) [1]. The anti-CD20 monoclonal antibody rituximab combined with chemotherapy, R-CHOP (rituximab with cyclophosphamide, doxorubicin, vincristine and prednisone), is a first-line standard regimen for DLBCL because of higher complete remission (CR) rates [2,3]. However, the main obstacle in treating patients with lymphoma is represented by the adverse events connected with immunochemotherapy [4]. Particularly for elderly or frail patients, the R-CHOP treatment plan mainly causes serious hematologic toxicity (myelosuppression, anemia, and thrombocytopenia) and other non-hematologic toxicity (neurotoxicity, gastrointestinal toxicity) in the administration of chemotherapeutic agents [5].

Myopenia, sarcopenia, and body composition measures such as lean body mass (LBM) have attracted more attention. Skeletal muscle loss is regarded as myopenia or sarcopenia. Both myopenia and sarcopenia represent muscle reduction and were highly prevalent age-related diseases [6,7,8]. The rate of sarcopenia in older adults (>70 years old) with colorectal cancer was more than 56%, which was related to higher susceptibility to chemotherapy toxicity [9]. Most anti-neoplastic drugs were administered according to body surface area (BSA) [4]. Women, on average, have a lower LBM per unit BSA than men. When BSA was used to calculate drug dosage, female colorectal cancer patients receiving adjuvant capecitabine had a higher incidence of dose-limiting toxicity (DLT) than male patients [10,11]. The anticancer drug dose is scaled to BSA, which considers weight and height, but no differences in body composition [12,13]. However, there was evidence that pharmacokinetics and drug toxicity were more associated with LBM. Body composition (adipose tissue and muscle mass) affects antineoplastic agents’ metabolism, clearance, and toxicity, and it can also predict the toxicity reactions of some chemotherapy regimens [14,15]. There were tremendous variations in LBM in patients with identical BSA. Therefore, as the only indicator of dose calculation, BSA was not enough to avoid serious toxicity [16]. The relative proportions of LBM and adipose tissue are also thought to be responsible for the variability in the patients’ toxicity. The total weight was composed of fat and lean. Fat may be the main distribution point of lipophilic drugs, while lean may be the main distribution point of non-lipophilic drugs [17]. LBM has a particular association of anticancer agents, and thus some of these authors proposed suggestions that LBM might be a better factor in estimating the distribution of drugs, but so far, conventional chemotherapy dosing has not been based on muscle tissue measures [18,19,20].

The use of widely used computed tomographic (CT) imaging to measure Lumbar 3 vertebral cross-section muscle index to evaluate the degree of sarcopenia has been the focus of recent oncology studies. It accurately and specifically quantifies muscle mass and distinguishes between adipose tissue and muscle tissue [21]. Skeletal muscle quantization ranges from −29 to +150 Hounsfield units (HU) [22]. Total skeletal muscle area (SMA, cm^2^) at the third lumbar (L3) cross-section measured by CT has been demonstrated to represent whole-body muscle mass [23]. The average HU of skeletal muscle means skeletal muscle density (SMD). Skeletal muscle index (SMI) was obtained by dividing SMA by height squared (m^2^) [18]. Skeletal muscle gauge (SMG) was first proposed by Weinberg et al. They multiplied SMI by SMD as SMG, which was more relevant to age than SMD or SMI alone [24]. The calculation formula of LBM is: LBM (kg) = L3 SMA (cm^2^) × 0.3 + 6.06 [11].

In DLBCL, there is increasing evidence that sarcopenia has an impact on intolerance and poor prognosis of the standard R-CHOP immunochemotherapy. However, they did not evaluate the association between adverse outcomes and body composition metrics, including SMG, SMI, SMD and LBM. With the increasing application of R-CHOP in clinical practice, we need to pay close attention to the prognosis and potential for treatment toxicity. Potential biomarkers that can predict treatment-related toxicity are urgently needed. We aim to use CT images to measure body compositions, and further assess the correlation between body composition indexes and R-CHOP immunochemotherapy adverse reactions and prognosis.

## 2. Materials and Methods

### 2.1. Participants

We collected the medical records of some patients who were diagnosed as DLBCL in the Harbin Medical University Cancer Hospital from January 2012 to December 2014. The pathological results of biopsy specimens combined with CT imaging examination supported the diagnosis in histology. The study was conducted in accordance with the Declaration of Helsinki and approved by the Ethics Committees of Harbin Medical University. All patients had to be at least 18 years old with CD20 positive DLBCL and received the standard five-drug chemoimmunotherapy combination R-CHOP treatment plan (rituximab 375 mg/m^2^ day 1, cyclophosphamide 750 mg/m^2^ day 1, doxorubicin 50 mg/m^2^ day 1, vincristine 1.4 mg/m^2^ maximum dose 2 mg day 1, and prednisone 100 mg/day on days 1 to 5), once every three weeks up to 6 cycles and no preventive application of G-CSF (Granulocyte Colony Stimulating Factor). However, the following patients were excluded: 1. Lack of evaluable CT image of the whole abdomen within 4 weeks before initial treatment; 2. Eastern Cooperative Oncology Group performance status score (ECOG) > 2 (Because patients with ECOG > 2 could not tolerate full-dose R-CHOP, they usually need to reduce their dose); 3. With second primary tumors.

### 2.2. Toxicity Grading and Follow-Up

The patients’ short or long-term adverse reactions during or after targeted therapy and chemotherapy were recorded by reviewing the documents of the patients or the telephone follow-up. Various toxicities were classified according to the National Cancer Institute Common Toxicity Criteria for Adverse Events (NCI-CTCAE) (Version 4.1) throughout the treatments [8]. The most frequent III–IV grade toxicity was hematological toxicity; there were also some non-hematological toxicities, for example, neurotoxicity, diarrhea, vomiting and other gastrointestinal toxicity. Hospitalization, dose reduction or termination of the treatment due to immunochemotherapy, and patients with high-grade adverse events, were the focus of our research. Secondly was the progression-free survival (PFS) and overall survival (OS). Patients were monitored on day 1, day 8 and day 15 of every cycle, or anytime they felt unwell, by physical examinations, hematology, and chemistry laboratory studies to assess and analyze the adverse events. Computed tomography (CT) scans were performed every 6–8 weeks during treatment to evaluate the change in the lesion. Patients with disease progression discontinued current treatment and immediately entered a follow-up phase.

### 2.3. CT-Based Body Composition Analysis

**Figure 1:**
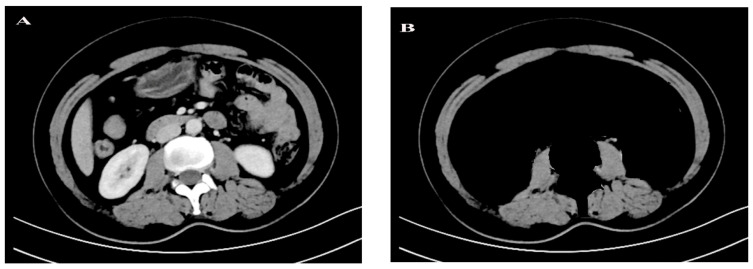
An example of abdominal computed tomography (CT) image. (**A**) The original CT image. (**B**) Skeletal muscle are quantified range of (−29 to + 150 Hounsfield units), and they were outlined along the epimysium that surrounded the muscles.

Single abdominal cross-sectional imaging of CT was considered as the preferred method for measuring and analyzing the whole-body composition of patients. We used SOMATOM Definition Flash CT-Siemens Healthineers (Germany) and GE Healthcare Optima (Sweden) for CT examination. The third lumbar vertebrae (L3) was selected as the standard marker to quantify skeletal muscles which were strongly associated with whole-body skeletal muscle mass. The subcutaneous and visceral adipose tissue and the skeletal muscles including psoas major, intercostal muscle, latissimus dorsi muscle and rectus abdominis were manually distinguished and outlined along the epimysium that surrounded the muscles by trained analysts. The highly accurate SMA (cm^2^) and the SMD from −29 to +150 Hounsfield Units (HU) could be obtained directly. Figure 1 shows an example of a CT image. Weight and height closest to the patients’ CT scan date before the initiation of therapy were selected from the medical records. Estimated of SMI and LBM were calculated using the validated formula as follows: SMI = (SMA (cm^2^))/(height^2^ (m^2^)), LBM (kg) = 0.3 × SMA (cm^2^) + 6.06. SMG was calculated by multiplying SMI and SMD. Furthermore, we represented the SMG units as arbitrary units (AU) for simplicity. Body mass index (BMI) was obtained by dividing the weight in kilograms (kg) by height squared in meters (m^2^).

### 2.4. Statistical Analysis

Descriptive statistics was adopted to describe the baseline characteristics of the patients. The correlation analysis data were calculated using binary logistic regression models, which was performed to identify whether lower body composition significantly increased the relative risk of toxicity by calculating the relative risk and 95% confidence interval ranges (95% CI).

Body compositions were identified as independent variables, toxicities were dependent variables, the ROC curve (receiver operating characteristic curve) was generated. The independent *t*-tests were used for the comparison of continuous variables between groups. DLBCL was divided into four stages, we used a scatter plot to evaluate the expression of body composition measures in each stage and calculated the *p*-value.

The comparisons between two groups of data were used by *t*-test. To further analyze the correlation between the two variables, we used a linear regression model to make a scatter plot representing the correlation of two variables.

Kaplan–Meier methods were performed to assess the impacts of body compositions parameters on PFS, OS and prognosis. We used Cox proportional hazards regression model for univariate and multivariate analysis. Statistical analysis was done using SPSS (SPSS for Windows, version 17.0, SPSS, Chicago, IL, USA) and GraphPad Prism 5 (GraphPad Software Inc., version 5.01, San Diego, CA, USA).

## 3. Results

### 3.1. Baseline Characteristics

Two hundred and one patients met our inclusion criteria, 43.3% were females. We compared patients’ baseline characteristics according to sex in Table 1. According to sex, significant differences could be found in body composition groups such as SMI, SMG, SMD, LBM (*p* < 0.001). No grade 5 toxicity (death) was recorded.

### 3.2. Body Composition Predicts Toxicity

Seventy-one percent (143/201) of patients occurred any grade 3–4 toxicity during or after immunochemotherapy. These toxicities were associated with poorer body composition. In Table 2, with 100 AU (arbitrary units) SMG reduction, the risk of any grade 3–4 toxicity was increased by 11% [RR (relative risk) = 1.11 [1.04, 1.18], *p* < 0.01]. For every 5 HU decreased in SMD, it was also increased in spite of being weakly influenced by 25% (RR=1.25 [1.02, 1.52], *p* < 0.05). For every 5 cm^2^/m^2^ decrease in SMI, it was increased by 34% (RR = 1.34 [1.12, 1.59], *p* < 0.01). With a 5 kg LBM reduction, it was increased by 35% (RR = 1.35 [1.13, 1.62], *p* < 0.01).

**Figure 2:**
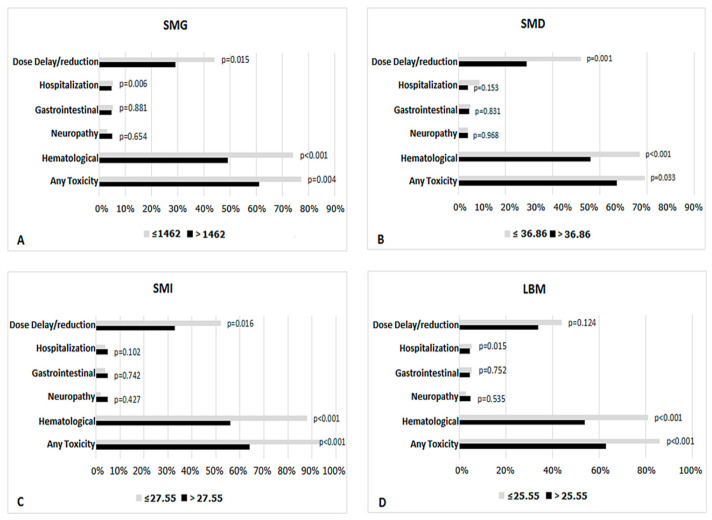
Risk of toxicity based on body composition. (**A**) SMG (skeletal muscle gauge), (**B**) SMD (skeletal muscle density), (**C**) SMI (skeletal muscle index), (**D**) LBM (lean body mass).

For further study, ROC curve and the area under the curve were utilized to analyze the relationship between the toxicity and body compositions (Figure S1). We identified the optimal cut-off values (least square root of sum of squared sensitivity plus squared 100-specificity) of body compositions as follows: SMG, 1462, SMD, 36.86, SMI, 27.55, LBM, 25.55 (Figure S2). Compared with patients with high body compositions, patients with low body compositions (below the cut-off value) suffered more frequent toxicity (Figure 2). We further explored the relationship between disease stage and body compositions (Figure S3). The median SMD and SMG based on stage I and II were higher compared to advanced patients (*p* < 0.001, *p* = 0.018). However, SMI and LBM did not show significant differences (Figure S3).

The dose of R-CHOP regimen was calculated according to BSA. For patients with DLBCL, BSA showed a weak correlation with SMG (r^2^ = 0.182, Figure S4). Patients with DLBCL had a very wide range of SMG, the retrospective data confirmed that patients with low SMG had a higher rate of toxicity. The range of data obtained by dividing the rituximab dose by SMG (dose/AU SMG) varied widely, and data analysis displayed that 0.605 rituximab mg/AU SMG was the optimal cut-off point for 3–4 grade toxicity. In Table 3, significant differences were discovered from the body composition groups including SMG, SMD, SMI and LBM, the mean values of the body composition above the cut-off point were less than that below (*p* < 0.001). Compared with the number of patients with dose/AU SMG ≤ 0.605 rituximab mg/AU SMG, the proportion of patients with dose/AU SMG > 0.605 rituximab mg/AU SMG showed to be higher in any 3–4 grade toxicity (*p* < 0.001), dose-limiting toxicity (*p* = 0.001), hematological toxicity (*p* < 0.001).

### 3.3. Body Composition Predicts Dose Delay/Reduction

Thirty-eight percent (76/201) of patients occurred dose delay/reduction (Table 2). For every 100 AU (arbitrary unit) SMG reduction, the risk of any grade 3–4 toxicity was increased by 10% [RR (relative risk) = 1.10 [1.04, 1.17], *p* < 0.01]. For every 5 HU decreased in SMD, it was also increased by 33% (RR = 1.33 [1.11, 1.59], *p* < 0.01). For every 5 cm^2^/m^2^ decrease in SMI, it was increased by 22% (RR = 1.22 [1.04, 1.44], *p* < 0.05). With 5 kg LBM reduction, it was increased by 20% (RR = 1.20 [1.01, 1.41], *p* < 0.05). Lower SMG, SMD, SMI and LBM were associated with an increasing risk of dose delay/reduction.

### 3.4. Body Composition Predicts Survival

**Figure 3:**
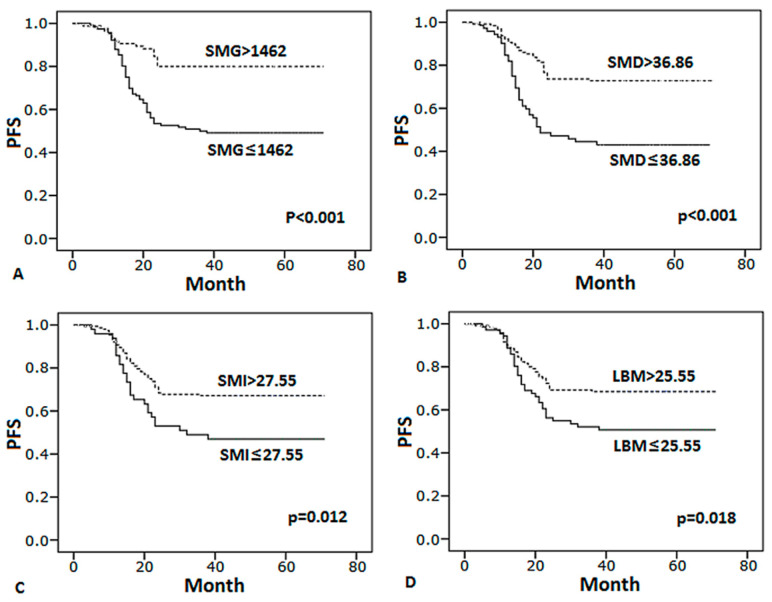
Kaplan–Meier curves of PFS for patients by body composition. (**A**) SMG (skeletal muscle gauge), (**B**) SMD (skeletal muscle density), (**C**) SMI (skeletal muscle index), (**D**) LBM (lean body mass).

**Figure 4:**
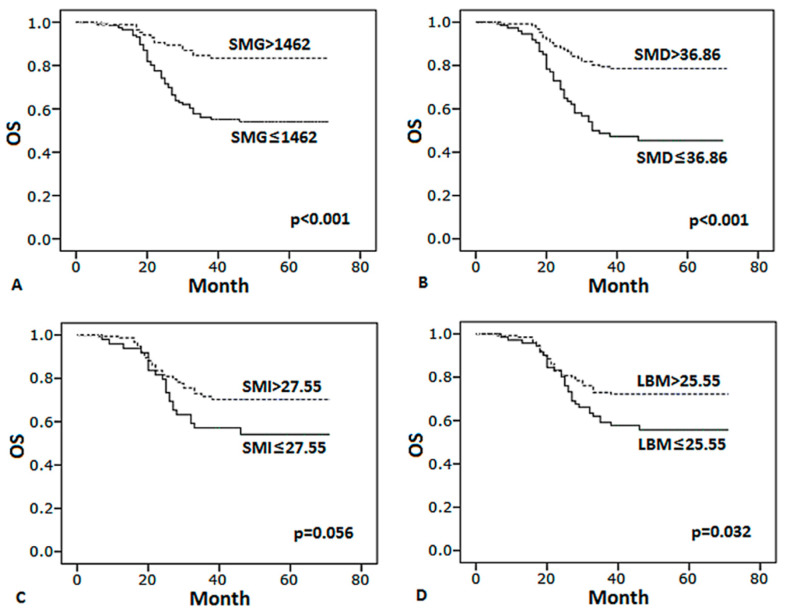
Kaplan–Meier curves of OS for patients by body composition. (**A**) SMG (skeletal muscle gauge), (**B**) SMD (skeletal muscle density), (**C**) SMI (skeletal muscle index), (**D**) LBM (lean body mass).

Compared with patients with high SMG, SMD, SMI and LBM, patients below the cut-off values had significantly worse PFS and OS (Figure 3 and Figure 4). In multivariate analysis (Table 4), SMG < 1462, III–IV stage, abnormal LDH levels, number of extranodal sites ≥ 2 remain identified as the significant independent predictors of worse PFS, SMG < 1462, LBM < 25.55, III–IV stage, number of extranodal sites ≥ 2 were predictive of worse OS. In conclusion, SMG was the best factor of body composition which was significantly associated with prognosis.

## 4. Discussion

Body composition is correlated with obesity, malnutrition, cachexia syndromes, metabolic syndrome frailty and so on. In particular, low skeletal muscle mass called sarcopenia or myopenia, has important implications of adverse outcomes in cancer patients. Lanic et al. found that sarcopenia was more common in elderly DLBCL patients with poor survival. They further proposed that the view that patients with sarcopenia have a poor prognosis should apply to the entire DLBCL population [25]. Meanwhile, sarcopenic obesity (obesity with depleted muscle mass) may predict functional status, chemotherapy toxicity and survival in malignant disease [17].

Some researchers have shown that body composition was associated to the treatment toxicity and prognosis of malignant diseases. Muscles radiation attenuation range from -29 to 150 HU on CT imaging, and SMD was reported as the average HU. Low SMD, in other words, meant muscle attenuation. Chu et al. observed that SMD was a novel and inexpensive prognostic marker independent of R-IPI (Revised International Prognostic Index) in DLBCL and follicular lymphoma [26]. SMI was known as the quantity of muscle, in gynecologic cancer, high-radiodensity SMI was the only indicator related to surgical complication and early mortality (<30 days). Low LBM reflected a loss of muscle, not weight loss, which was indicative of pathological metabolic states, such as severe malnutrition or cachexia. As the product of SMD and SMI, SMG was firstly paid close attention to by Weinberg et al., notably, that the age-related changes were more relevant to SMG [24]. Later, Shlomit et al. showed that low SMG has an effect on grade 3–4 hematological toxicities, gastrointestinal toxicities and hospitalizations in early breast cancer patients receiving doxorubicin-cyclophosphamide (AC)-taxane (T) chemotherapy regimens [27].

This is the first study on the correlation between SMI, SMD, SMG, LBM with treatment toxicity and prognosis for DLBCL patients receiving R-CHOP immunochemotherapy as initial treatment. Our findings were consistent with a recent series of publications that showed the connection between body composition and toxicity. We found that the measures of muscle metrics are more important than BSA and BMI. According to binary logistic regression models analysis, we found that the proportions of the risk of toxicity (any grade 3–4 toxicity, hematological toxicity, hospitalization) increased after the reduction of SMG, SMD, SMI and LBM in varying degrees. In addition, based on ROC analysis, we found that low SMG, SMI, SMD or LBM are associated with any grade 3–4 toxicity and grade 3–4 hematological toxicity. Of note, cut-off points of SMG, SMI and SMD were also useful predictors of dose delay and reduction. We further discovered that stage III and IV DLBCL patients had lower body composition. Furthermore, we demonstrated that patients with higher SMG, SMI, SMD or LBM had a significantly longer PFS and OS. However, in multivariate analyses, only SMG was significantly related to prognosis.

In oncology, chemotherapy doses were usually calculated according to BSA [13]. Whereas, the BSA formula only considers weight and height, ignoring whether weight is associated with increased muscle tissues or adipose tissues, due to BSA being weakly correlated with SMG [17]. Therefore, the use of BSA to individualized medication has also been questioned [13]. Prado et al. reported that colorectal cancer patients may have a low LBM relative to their BSA, suggesting that normalization of 5-fluorouracil dose to LBM (i.e., FMM) may be a better method of individualized administration and prevention of excessive toxicity than conventional BSA. Raafi et al. also proved that low LBM predicts neuropathy and toxicity in patients using the BSA to calculate the dose of the FOLFOX-based regimens [11].

In our study, the difference in SMG made high variation of Rituximab dose per AU SMG [range (0.231–12.915) mg/AU SMG]. Of 201 patients, most patients with low SMG levels suffered from toxicity, and their rituximab/SMG dose was higher than patients with high SMG levels. In evaluating the pharmacokinetics of rituximab, it was thought that weight gain or muscle mass gain in male increased rituximab clearance, which was associated with decreased rituximab efficacy [28]. The survival advantage of female was confirmed in subsequent retrospective data [29]. Sex differences in body composition are well known, such as SMG, SMD, SMI and LBM in this paper. It is worth considering whether patients treated in this way could tolerate and benefit from higher doses. Notably, this cut-off point may help to identify patients at high risk of toxicity, and we will explore that in future experiments.

Furthermore, by understanding the effects of body component and muscular decline on cancer patients, we believe it is worth further investigating whether timely interventions to increase muscle mass would improve sarcopenia and better lymphoma prognosis.

Limitations of this study include few studies about SMG; hence, more studies are encouraged to confirm the value of SMG in the future. Another potential limitation is the inherent bias introduced by retrospective analysis. Serum samples were also not uniformly available in order to evaluate rituximab or other drugs’ pharmacokinetics across these patients because of its retrospective nature as well. The final limitation is the limited number of patients with DLBCL, for this reason we did not get the different cut-off points of SMG, SMI, SMD and LBM by sex or age.

DLBCL is one of the most common lymphoma worldwide, and immunochemotherapy regimens are the main useful treatments for DLBCL. We have verified that body composition can predict toxicities and prognosis of patients with DLBCL. Moreover, we underlined the necessity for some interventions to improve unfavorable body composition so as to improve patients’ prognosis and decrease treatment-related toxicity potentially. Applying skeletal muscle mass assessment using available CT scans and imaging software to the clinic may be certified as one factor in IPI in the future. However, more trials including pharmacokinetics measures of the biologic or chemotherapeutic agents are needed to develop novel dosing strategies in patients with malignant diseases.

## 5. Conclusions

This manuscript, the largest study to date, assessed the effects of a variety of body composition measures on the toxicity and prognosis of chemotherapy in DLBCL patients undergoing immunochemotherapy. Our results showed that SMI, SMD, SMG and LBM could predict chemotherapy adverse outcomes and prognosis in DLBCL. However, in multivariate analyses, only higher SMG was a significant predictor of better outcomes and extended survival. Our findings suggest that measurements of body composition obtained from conventional CT images may play a role in individualizing administration of antitumor drugs in the future.

## Figures and Tables

**Table 1:** Baseline characteristics of diffuse large B-cell lymphoma by sex.

Characteristic	All Patients (*n* = 201)	Sex		*p*-Value
		Male (*n* = 114)	Female (*n* = 87)	
Age, years	56.9 ± 11.4	57.7 ± 11.3	56 ± 11.4	0.307
Age < 65 years, *n* (%)	147 (73)	80 (70)	67 (77)	0.605
Age ≥ 65 years, *n* (%)	54 (27)	34 (30)	20 (23)	0.599
Disease stage, *n* (%)				0.682
I	16 (7.9)	5 (4.3)	11 (12.6)	
II	72 (35.8)	43 (37.7)	29 (33.3)	
III	77 (38.3)	49 (43)	28 (32.1)	
IV	36 (17.9)	17 (15)	19 (21.8)	
LDH				0.512
normal	107 (53.2)	63 (55.3)	44 (50.6)	
abnormal	94 (46.8)	51 (44.7)	43 (49.4)	
IPI score				0.842
0–2	164 (81.6)	95 (83.3)	68 (78.2)	
3–4	37 (18.4)	19 (16.7)	19 (21.8)	
BMI, kg/m^2^	23.1 ± 3.1	23.4 ± 3.1	22.7 ± 3.2	0.132
BMI group, *n* (%)				0.132
BMI < 20	30 (14.9)	14 (12.3)	15 (17.2)	
BMI 20–24.9	112 (55.7)	60 (52.6)	52 (61)	
BMI > 25	59 (29.4)	41 (35.1)	19 (21.8)	
Skeletal muscle area, cm^2^	95.4 ± 29.7	111.6 ± 25.2	74.1 ± 20.2	<0.001
Skeletal muscle gauge, AU	1350.7 ± 524	1583 ± 472.8	1046.3 ± 423.6	<0.001
Skeletal muscle index, cm^2^/m^2^	34 ± 9.2	38.2 ± 8.3	28.6 ± 7.3	<0.001
Skeletal muscle radiodensity, HU	38.6 ± 8.5	40.9 ± 6.7	35.5 ± 9.5	<0.001
Estimated lean body mass, kg	30.4 ± 8.9	35.3 ± 7.6	24 ± 6.0	<0.001

Data are shown as *n* (%) or mean ± SD. Abbreviations: BMI, body mass index; HU, Hounsfield unit; IPI, international prognostic index; LDH, lactate dehydrogenase; SD, standard deviation.

**Table 2:** Relative risk (RR) (95% Confidence Interval) of toxicity for body composition measures.

	Any Grade 3–4 Toxicity *n* = 143	Any Grade 3–4 Hematological Toxicity *n* = 128	Any Grade 3–4 Neurotoxicity *n* = 8	Any Grade 3–4 GI Toxicity *n* = 10	Hospitalization *n* = 12	Dose Delay/Reduction *n* = 76
SMG 100 AU decrease	1.11 (1.04, 1.18) **	1.15 (1.08, 1.23) **	0.99 (0.86, 1.13)	1.01 (0.9, 1.14)	1.23 (1.08, 1.41) **	1.10 (1.04, 1.17) **
SMD 5 HU decrease	1.25 (1.02, 1.52) *	1.42 (1.16, 1.74) **	0.89 (0.56, 1.39)	0.9 (0.6, 1.34)	1.58 (1.17, 2.14) **	1.33 (1.11, 1.59) **
SMI 5 cm^2^/m^2^ decrease	1.34 (1.12, 1.59) **	1.44 (1.21, 1.71) **	0.98 (0.67, 1.44)	1.11 (0.78, 1.57)	1.78 (1.23, 2.57) **	1.22 (1.04, 1.44) *
LBM 5 kg decrease	1.35 (1.13, 1.62) **	1.46 (1.22, 1.75) **	0.96 (0.64, 1.43)	1.16 (0.81, 1.68)	1.94 (1.27, 2.95) **	1.20 (1.01, 1.41) *
BMI 1 kg/m^2^ decrease	1.06 (0.96, 1.17)	1.06 (0.97, 1.17)	1.04 (0.83, 1.31)	0.94 (0.77, 1.15)	1.12 (0.92, 1.36)	0.96 (0.88, 1.05)
visceral adipose tissue density 1 unit decrease	1.01 (0.98, 1.03)	1.00 (0.97, 1.02)	1.01 (0.95, 1.08)	1.03 (0.96, 1.1)	1.03 (0.97, 1.09)	1.01 (0.99, 1.04)
visceral adipose tissue area 1 unit decrease	1.00 (1, 1.01)	1.01 (1, 1.01)	1.00 (0.98, 1.02)	0.99 (1, 1.01)	1.00 (0.99, 1.01)	1.00 (0.99, 1)
subcutaneous adipose tissue density 1 unit decrease	1.02 (1, 1.04)	1.02 (1, 1.04)	0.99 (0.95, 1.03)	1.02 (1, 1.04)	1.02 (0.96, 1.08)	0.99 (0.97, 1.02)
subcutaneous adipose tissue area 1 unit decrease	1.00 (0.99, 1)	1.00 (0.99, 1)	1.00 (0.99, 1.02)	1.01 (1, 1.03)	1.00 (0.99, 1.01)	1.00 (0.99, 1.003)

* *p* < 0.05; ** *p* < 0.01; Abbreviations: BMI, body mass index; GI, gastrointestinal; HU, Hounsfield unit; LBM, lean body mass; SMD, skeletal muscle radiodensity; SMG, skeletal muscle gauge; SMI, skeletal muscle index.

**Table 3:** Comparison of patients who received Rituximab per AU SMG.

Variables	R-CHOP Regimens		*p*-Value
	Rituximab/SMG ≤0.605 mg/AU	Rituximab/SMG >0.605 mg/AU	
Number of patients	142	59	
Sex, n (%)			**<0.001**
Male	96(67.6)	18(30.5)	
Female	46(32.4)	41(69.5)	
Age, years, mean ± SD	54.3 ± 10.8	63.5 ± 10.0	**<0.001**
Disease stage, *n* (%)			**0.012**
I vs. II	70(49.3)	18(30.5)	
III vs. IV	72(50.7)	41(69.5)	
BMI, kg/m^2^, mean ± SD	23.2 ± 3.1	23.0 ± 3.3	0.675
BMI group, *n* (%)			
<20	19(13.4)	10(17)	0.514
20–24.9	77(54.2)	32(54.2)	0.857
25–29.9	46(32.4)	17(28.8)	0.762
Skeletal muscle area, cm^2^, mean ± SD	106.8 ± 25.6	67.9 ± 18.7	**<0.001**
Skeletal muscle gauge, mean ± SD	1599 ± 383.9	745 ± 251.8	**<0.001**
Skeletal muscle index, cm^2^ /m^2^	37.7 ± 7.7	25.1 ± 5.9	**<0.001**
Skeletal muscle radiodensity, HU, mean ± SD	42.4 ± 5.3	29.2 ± 7.3	**<0.001**
Estimated lean body mass, kg, mean ± SD	33.9 ± 7.7	22.2 ± 5.6	**<0.001**
Rituximab (mg/AU SMG)	0.43 ± 0.09	1.22 ± 1.83	**0.002**
Any 3–4 grade toxicity, Number (%)	92(64.8)	51(86.4)	**<0.001**
Dose-limiting toxicity	43(30.3)	33(55.9)	**0.001**
Neuropathy	6(4.2)	2(3.4)	0.784
Hematological toxicity	78(54.9)	50(84.7)	**<0.001**
GI toxicity	7(4.9)	3(5.1)	0.963
Hospitalization	5(3.5)	7(11.9)	0.069

Data are shown as *n* (%) or mean ± SD. Abbreviations: BMI, body mass index; ECOG, Eastern Cooperative Oncology Group; GI, gastrointestinal; HU, Hounsfield unit; SD, standard deviation. Compared with the number of patients with dose/AU SMG ≤ 0.605 rituximab mg/AU SMG, the proportion of patients with dose/AU SMG > 0.605 rituximab mg/AU SMG showed to be higher in any 3–4 grade toxicity (*p* < 0.001), dose-limiting toxicity (*p* = 0.001), hematological toxicity (*p* < 0.001) (bold numbers).

**Table 4:** Univariate and multivariate analysis for OS and PFS outcomes.

	OS			PFS		
	HR	CI	*p*-Value	HR	95% CI	*p*-Value
**Univariate Analysis**						
Age, years						
>60 vs. ≤60	1.663	1.030–2.685	**0.038**	1.452	0.925–2.28	0.105
Disease stage						
III–IV vs. I–II	4.082	2.225–7.489	**<0.001**	3.846	2.212–6.686	**<0.001**
LDH level						
Elevated vs. Normal	2.425	1.471–4.000	**0.001**	2.67	1.661–4.292	**<0.001**
Number of extranodal sites						
2–4 vs. 0–1	3.417	1.942–6.012	**<0.001**	3.143	1.824–5.416	**<0.001**
IPI score						
3–4 vs. 0–2	3.492	2.122–5.748	**<0.001**	3.020	1.875–4.864	**<0.001**
SMG						
≤1462 vs. >1462	3.313	1.837–5.976	**<0.001**	3.126	1.820–5.370	**<0.001**
SMD						
≤36.86 vs. >36.86	3.141	1.925–5.125	**<0.001**	2.676	1.700–4.213	**<0.001**
SMI						
≤27.55 vs. >27.55	1.645	0.987–2.74	0.056	1.837	1.143–2.953	**0.012**
LBM						
≤25.55 vs. >25.55	1.674	1.035–2.707	**0.036**	1.724	1.098–2.708	**0.018**
Rituximab/SMG mg/AU						
>0.605 vs. ≤0.605	3.436	2.123–5.562	**<0.001**	3.102	1.973–4.879	**<0.001**
**Multivariate analysis**						
Disease stage						
III–IV vs. I–II	3.027	1.522–6.021	**0.002**	3.250	1.770–5.967	**<0.001**
LDH level						
Elevated vs. Normal	1.522	0.833–2.779	0.172	2.017	1.167–3.487	**0.012**
Number of extranodal sites						
2–4 vs. 0–1	2.381	1.177–4.816	**0.016**	2.565	1.329–4.952	**0.005**
Age, years						
>60 vs. ≤60	1.028	0.497–2.127	0.940	-	-	-
IPI score						
3–4 vs. 0–2	1.071	0.430–2.664	0.883	1.402	0.730–2.690	0.310
SMG						
≤1462 vs. >1462	2.655	1.218–5.787	**0.014**	2.889	1.401–5.959	**0.004**
SMD						
≤36.86 vs. >36.86	1.113	0.514–2.409	0.787	1.105	0.541–2.255	0.784
SMI						
≤27.55 vs. >27.55	-	-	-	1.217	0.521–2.842	0.651
LBM						
≤25.55 vs. >25.55	2.145	1.194–3.853	**0.011**	2.151	0.986–4.695	0.054
Rituximab/SMG mg/AU						
>0.605 vs. ≤0.605	2.115	0.956–4.676	0.064	1.988	0.903–4.376	0.088

Abbreviations: CI, confidence interval; HR, hazard ratio; LBM, lean body mass; LDH, lactic dehydrogenase; OS, overall survival; PFS, progression-free survival; SMD, skeletal muscle radiodensity; SMG, skeletal muscle gauge; SMI, skeletal muscle index. SMG was the best factor of body composition which was significantly associated with prognosis (bold numbers).

## Data Availability

The data are not publicly available due to privacy and confidentiality.

## Supplementary Materials

The following are available online at https://www.mdpi.com/1718-7729/28/2/126/s1, Figure S1: ROC curve (receiver operating characteristic curve) and area AUC (area under the curve) for the correlation between body composition and immunochemotherapy toxicity. (A) SMG (skeletal muscle gauge), (B) SMD (skeletal muscle density), (C) SMI (skeletal muscle index), (D) LBM (lean body mass). Figure S2: A histogram showing patient distribution. SMG (skeletal muscle gauge), SMD (skeletal muscle density), SMI (skeletal muscle index), LBM (lean body mass). Figure S3: The expression of body composition in different stages. (A) SMG (skeletal muscle gauge), (B) SMD (skeletal muscle density), (C) SMI (skeletal muscle index), (D) LBM (lean body mass). Figure S4: Pearson correlation between BSA (body surface area) and SMG (skeletal muscle gauge).
