# Supplementary material for: Body Composition as a Predictor of Toxicity and Prognosis in Patients with Diffuse Large B-Cell Lymphoma Receiving R-CHOP Immunochemotherapy
Source: Curr Oncol. 2021 Mar 23;28(2):1325–37. doi: 10.3390/curroncol28020126 (PMC8025815; doi:10.3390/curroncol28020126)
Supplement: Supplementary file 1 [file curroncol-28-00126-s001.pdf]

Current Oncology

MDPI

Article

Body Composition as a Predictor of Toxicity and Prognosis in Patients with Diffuse Large B-Cell Lymphoma Receiving R-CHOP Immunochemotherapy

Jiaxun Guo, Panpan Cai, Pengfei Li, Cong Cao, Jing Zhou, Lina Dong, Yan Yang, Qijia Xuan, Jingxuan Wang and Qingyuan Zhang

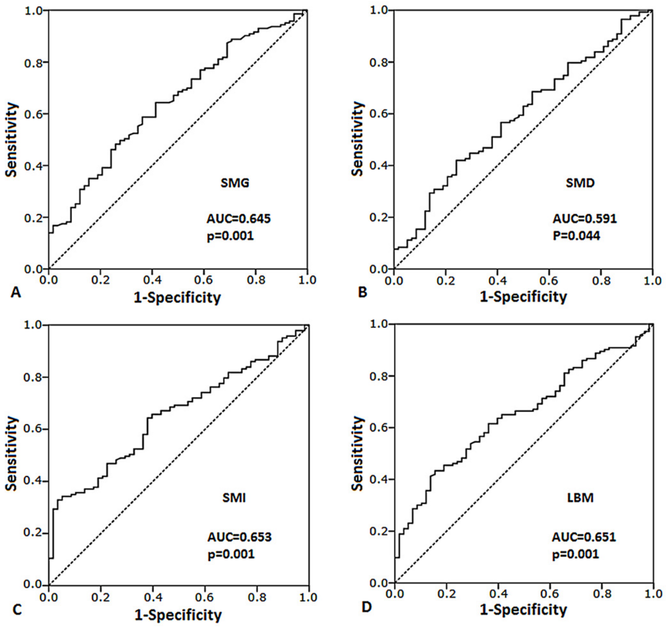

Figure S1. ROC curve (receiver operating characteristic curve) and area AUC (area under the curve) for the correlation between body composition and immunochemotherapy toxicity. (**A**) SMG (skeletal muscle gauge), (**B**) SMD (skeletal muscle density), (**C**) SMI (skeletal muscle index), (**D**) LBM (lean body mass).

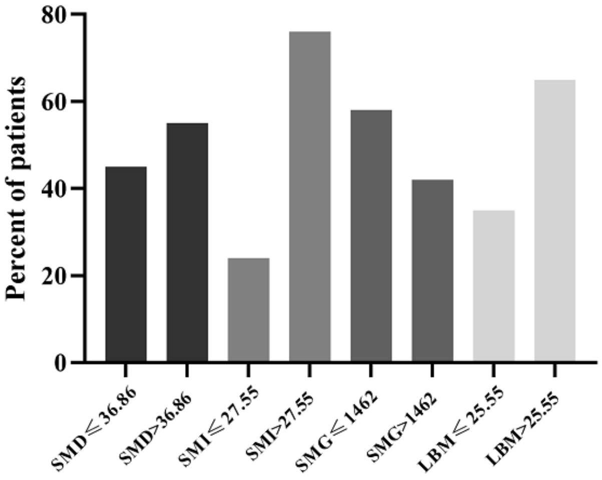

Figure S2. A histogram showing patient distribution. SMG (skeletal muscle gauge), SMD (skeletal muscle density), SMI (skeletal muscle index), LBM (lean body mass).

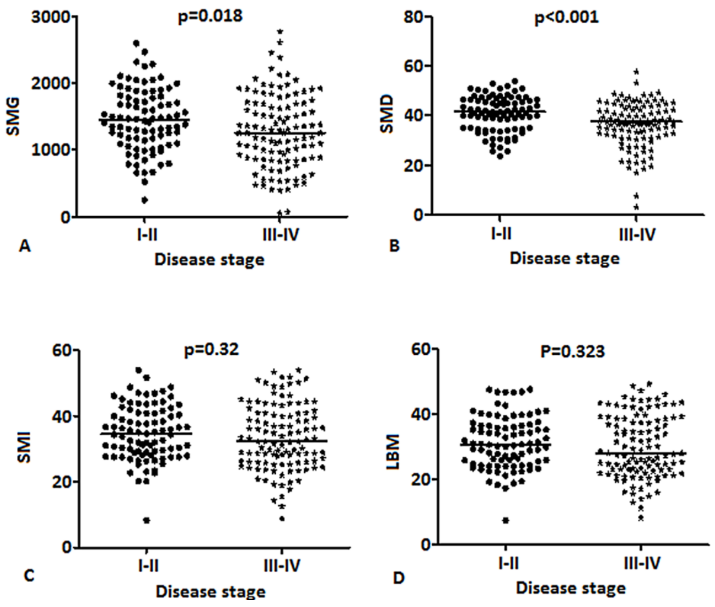

Figure S3. The expression of body composition in different stages. (A) SMG (skeletal muscle gauge), (B) SMD (skeletal muscle density), (C) SMI (skeletal muscle index), (D) LBM (lean body mass).

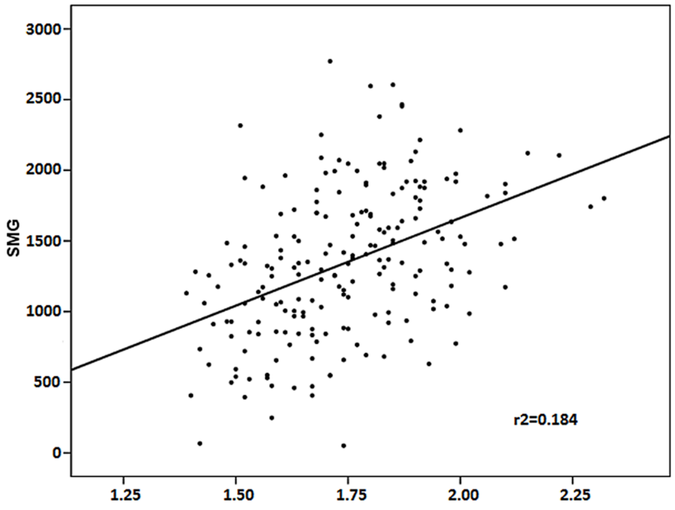

Figure S4. Pearson correlation between BSA (body surface area) and SMG (skeletal muscle gauge).
